# Oxidative Stress Induces Telomere Dysfunction and Senescence by Replication Fork Arrest

**DOI:** 10.3390/cells8010019

**Published:** 2019-01-03

**Authors:** Elisa Coluzzi, Stefano Leone, Antonella Sgura

**Affiliations:** Department of Science, University of Rome “Roma TRE”, Viale Guglielmo Marconi, 446, 00146 Rome, Italy; elisa.coluzzi@uniroma3.it (E.C.); stefano.leone@uniroma3.it (S.L.)

**Keywords:** telomere, oxidative stress, replication fork, epigenetics

## Abstract

Oxidative DNA damage, particularly 8-oxoguanine, represents the most frequent DNA damage in human cells, especially at the telomeric level. The presence of oxidative lesions in the DNA can hinder the replication fork and is able to activate the DNA damage response. In this study, we wanted to understand the mechanisms by which oxidative damage causes telomere dysfunction and senescence in human primary fibroblasts. After acute oxidative stress at telomeres, our data demonstrated a reduction in TRF1 and TRF2, which are involved in proper telomere replication and T-loop formation, respectively. Furthermore, we observed a higher level of γH2AX with respect to 53BP1 at telomeres, suggesting a telomeric replication fork stall rather than double-strand breaks. To confirm this finding, we studied the replication of telomeres by Chromosome Orientation-FISH (CO-FISH). The data obtained show an increase in unreplicated telomeres after hydrogen peroxide treatment, corroborating the idea that the presence of 8-oxoG can induce replication fork arrest at telomeres. Lastly, we analyzed the H3K9me3 histone mark after oxidative stress at telomeres, and our results showed an increase of this marker, most likely inducing the heterochromatinization of telomeres. These results suggest that 8-oxoG is fundamental in oxidative stress-induced telomeric damage, principally causing replication fork arrest.

## 1. Introduction

Reactive oxygen species (ROS) can be produced by exogenous or endogenous factors. These molecules are highly reactive and unstable and can damage different cellular components, such as proteins, lipids and DNA [1]. Oxidative damage constitutes the majority of DNA damage in human cells, appearing as an oxidized base, a sugar modification, a DNA or protein crosslink or a DNA strand break [2,3,4,5]. The marker 8-oxoguanine (8-oxoG) has been widely used to detect oxidative damage. This compound is highly mutagenic because, generally, the presence of an unrepaired oxidized base in the DNA was bypassed by DNA polymerase, which predominantly introduced dAMP opposite 8-oxoG, inducing a G:C-T:A transversion mutation that results in genomic instability. Furthermore, the oxidation of guanine could also occur in the dNTP pool; in this case, the incorporation of the 8-oxoG dGMP opposite to the dC or dA on the template strand could induce an A:T-C:G transversion during the subsequent replication [6]. Oxidative stress, inducing base modifications and single strand breaks (SSBs), could interfere with the replication machinery and is able to activate the DNA damage response (DDR) through different checkpoint pathways that activate specific proteins [7,8]. Among these proteins, ATR stabilizes the stalled fork, promotes cell-cycle arrest and activates DDR, followed by the phosphorylation of H2AX and RPA, which protect ssDNA [9,10]. γH2AX is observed immediately at stalled replication forks and spreads to nearby regions. This evidence suggests that the phosphorylation of H2AX is not only activated after the DDR, but also in the case of stalled replication forks [9]. The colocalization of γH2AX or 53BP1 (both markers of DNA damage, in particular double strand breaks, DSBs) with the end of chromosomes, namely telomeres, suggests telomeric damage by telomere dysfunction-induced foci (TIFs) [11].

Telomeres are essential for chromosome and genome stability. In humans, non-coding repetitive TTAGGG sequences, histone modification marks and telomeric binding proteins constitute the telomere structure [12,13,14]. Telomeres became shorter at each cell division before reaching a state of replicative senescence, called the Hayflick limit, in which the cell is no longer able to divide [15]. However, the senescence phenotype could be induced by telomeric-independent triggers. Among these triggers is the cellular accumulation of ROS, which in turn induces excessive DNA damage and could activate a stress-response program known as stress-induced premature senescence (SIPS), characterized by permanent cell cycle arrest, enhanced cell size and an increased number of cells positive for senescence-associated β-galactosidase activity (SA-β-Gal) [16,17,18,19,20].

At the telomeric level, 8-oxoG represents the most frequent oxidative DNA lesion because of the high level of guanine in the sequence. Furthermore, due to the telomeric heterochromatin state, the oxidative telomeric damage is less efficiently repaired compared to the oxidative damage widespread in the genome [21]. All of these features make telomeres the preferential sequence damaged by oxidative stress [22,23]. Normally, telomeres are stabilized by shelterin proteins, which are dynamic structural components of the telomere. These proteins interact with other associated DNA factors to remodel and change the structure of telomeric DNA, and do not lose their main function of protection [13]. Telomeric proteins have a remarkable specificity for telomeric sequences TTAGGG. In particular TRF1 and TRF2 directly bind duplex telomere repeats as homodimers, playing the main role in the telomere structure [13,24]. It has been shown that oxidative stress, by 8-oxoG modification, is able to change the capability of TRF1 and TRF2 to bind telomeric sequences, as demonstrated in oligomers [23].

Although these two proteins have similar functional domains, their cellular functions are different: TRF2 is essential for the formation of the t-loop and for the protection of the 3’ G-overhang, whereas TRF1 binds the circular segment of the duplex and is essential for the efficient replication of telomeres, preventing the stall of the replication fork and the consequent telomere breakages [25,26,27,28]. In fact, previous studies have demonstrated that the deletion of TRF1 induces the formation of multitelomeric signals (MTS), referred to as fragile telomeres, and a fragile site is commonly due to a hinderance in the replication machinery [27].

However, telomere function is not only maintained by a minimum telomere length and an efficient shelterin complex but also by a histone modification marks that define the heterochromatic telomeric structure [13,29]. It was hypothesized that there is cooperation between not only the shelterin complex and heterochromatin factors, but also DNA replication and the chromatin assembly [30,31]. The binding of the shelterin complex, in turn, is affected by the telomeric chromatin context and could influence the protected or deprotected state of telomeres [14,32]. Commonly, telomeres are associated with characteristic repressive histone modification marks, H3K9me3 and H4K20me, and with specific proteins, including HP1, which is recruited to chromatin by its affinity for trimethylated H3K9 (H3K9me3), indicating that telomeres are assembled into heterochromatin domains [33]. These telomeric epigenetic modifications are fundamental for the regulation of telomere length and the structural integrity of the closed/silenced chromatin state [34]. Very little is known about the oxidative stress and epigenetic changes at telomeres; however, it has been shown that global DNA methylation and methylated repressive histone marks, such as H3K4me3 and H3K9me3, can increase after oxidative stress [35,36,37].

Furthermore, at the genomic level, epigenetic changes have been associated with replication fork arrest but in this case, no information is available on the relationship between epigenetic changes and replication stress at telomeres [38,39].

In our previous study we demonstrated, after acute oxidative stress, the persistence of telomeric oxidative damage (8-oxoG), which causes telomere shortening and increases chromosome instability due to telomeric fusion [21]. However, in the present study our question was: how does 8-oxoG induce telomere shortening/dysfunction? Because very little is known about the effects of oxidative stress on telomere replication and telomeric epigenetic changes, to understand the mechanisms that lead to telomere damage after hydrogen peroxide treatment, we analyzed the shelterin proteins TRF1 and TRF2, the activation of the DDR pathway, replication fork arrest and epigenetic modifications of telomeres, particularly the amount of H3K9me3 heterochromatin mark. Our results demonstrate that for telomeres, replication fork arrest, epigenetic changes for H3K9me3 and the reduction of TRF1 and TRF2 occur after oxidative stress, and our hypothesis is that persistent 8-oxoG is the main player in these modifications.

## 2. Materials and Methods

### 2.1. Cell and Culture Conditions

Human primary fibroblasts MRC-5, derived from fetal human lung primary culture (ECACC, Salisbury, UK), were grown in modified Eagle’s medium (MEM) (Euroclone, Pero, Milano, Italy) supplemented with 10% fetal bovine serum (Euroclone, Pero, Milano, Italy), 100 units/mL penicillin and streptomycin 10 µg/mL (Biological Industries, Israel), 1% L-Glutamine and 1% non-essential amino acids (Euroclone, Italy), at 37 °C in 95% air and 5% CO_2_ incubator. According to the previous experimental design, sub confluent cells, at maximum PD 30, were treated with H_2_O_2_ (10 vol–3%), in a complete medium, at the final concentration of 100 and 200 μM for 1 h at 37 °C and then trypsinized and seeded at different concentration depending on the protocols [21]. Cells examined after treatment with H_2_O_2_ were compared to parallel cultured control cells grown in the medium without H_2_O_2_.

### 2.2. Chromatin Immunoprecipitation (ChIP)

After 1 h treatment with 100 µM and 200 µM H_2_O_2_, cells were trypsinized and seeded in a petri dish and left to grown in a complete medium up to 48 h after treatment. For ChIP analysis, 4 × 10^6^ cells were used per condition. Formaldehyde, at a final concentration of 1%, was added directly to the medium for 15 min at room temperature (RT) on a shaking platform. Glycine, to a final concentration of 0.150 M, was added to the medium to stop the cross-link. Cells were then washed twice in cold phosphate buffered saline (PBS) containing protease inhibitors, collected and lysed at the density of 20 × 10^6^/mL for 10 min at 4 °C in 1% SDS, 50 mM Tris-HCl pH 8.0 and 10 mM EDTA containing protease inhibitors. Lysate were sonicated (Bandelin SONOREX RK 100H, Bandelin electronic GmbH & Co. KG, Berlin, Germany) to obtain chromatin fragments <1 kb and centrifuges for 15 min a RT. Chromatin was diluted 1:10 in a buffer containing 1.1% Triton X-100, 0.01% SDS, 1.2 mM EDTA, 167 mM NaCl, 16.7 mM Tris-HCl pH 8.0, protease inhibitors and salmon sperm DNA-Protein A 50% agarose slurry (Santa Cruz Biotechnology, Inc., Dallas, TX, USA). Chromatin fragments were incubated, overnight at 4 °C on a rotating platform with different antibodies: TRF1 (Santa Cruz Biotechnology), TRF2 (Cell Signaling Technology, Danvers, MA, USA), H3 (abcam, Cambridge, UK), H3K9me3 (Millipore, Burlington, MA, USA), pre-immune serum (Jackson ImmunoResearch Laboratories, Inc, Baltimore Pike, PA, USA). Salmon sperm DNA-protein A agarose beads were then added and the incubation continued for 1 h at 4 °C. Immunoprecipitated pellets were washed one time for 3 min with different buffers: Low salt buffer (0.1% SDS, 1% Triton X-100, 2 mM EDTA, 20 mM Tris-HCl pH 8.0 and 150 mM NaCl); high salt buffer (0.1% SDS, 1% Triton X-100, 2 mM EDTA, 20 mM Tris-HCl pH 8.0 and 500 mM NaCl); LiCl Buffer (0.25 M LiCl, 1% Nonidet P-40, 1% sodium deoxycholate, 1 mM EDTA and 10 mM Tris-HCl, pH 8.0); and two washes whit TE (10 mM Tris-HCl pH 8.0 and 1 mM EDTA). Chromatin was eluted from the beads twice by incubation with 250 µL of 1% SDS, 50 mM NaHCO_3_ for 15 min at RT with rotation. After adding 20 µL of 5 M NaCl, crosslinks were reversed by incubation o.n. at 65 °C. Samples were supplemented with 20 µL of 1 M Tris-HCl pH 6.5, 10 µL of 0.5 M EDTA, 20 µg of RNase A and 40 µg of proteinase K and were incubated for 1 h at 45 °C. DNA was then recovered by phenol-chloroform extraction and ethanol precipitation, slot-blotted into a Hybond N+ membrane (Amersham Pharmacia Biotech, Milano, Italy) and hybridized with a telomeric probe obtained from a plasmid containing 1.6 kb of TTAGGG repeats (kindly provided by M. Blasco, Spanish National Cancer Centre-CNIO). We quantified the signal using ImageJ software. For total DNA samples, aliquots corresponding to a 1:10 dilution of the amount of lysate used in the immunoprecipitations were processed along with the rest of the samples during the crosslink reversal step. We calculated the amount of telomeric DNA immunoprecipitated in each ChIP based on the signal relative to the corresponding total telomeric DNA input signal. We represented the ChIP values as a percentage of the total input telomeric DNA, thus correcting for differences in the number of telomere repeats [40]. At least three independent experiments were performed for each antibody used.

### 2.3. γH2AX and 53BP1 Immunofluorescence Staining

After 1 h treatment with two doses of hydrogen peroxide, cells were trypsinized and seeded on a glass in a petri dish at different density. The slides were fixed with 4% paraformaldehyde (Sigma Aldrich, St. Louis, MO, USA), permeabilized in 0.2% Triton X-100 and blocked in PBS/BSA 1% for 30 min at RT. Slides were incubated with a mouse mono-clonal anti-phospho-histone H2AX antibody (Millipore) or an anti-53BP1 antibody (Novus Biologicals, Littleton, CO, USA) overnight at 4 °C, washed in PBS/BSA 1% and then exposed to the secondary Alexa 488-labeled donkey anti-mouse antibody (Invitrogen, Life Technologies, Carlsbad, CA, USA) for γH2AX and Alexa Fluor 488 labeled goat anti-rabbit (Invitrogen, Life Technologies, Carlsbad, CA, USA) for 53BP1, for 1 h at 37 °C. After washes in PBS/BSA 1% DNA were counterstained with DAPI (Sigma Aldrich) in Vectashield (Vector Laboratories, Burlingame, CA, USA). Cells were analyzed with fluorescence microscopy using an Axio Imager Z1 microscope (Carl Zeiss, Oberkochen, Germany) equipped with the Metacyte module of the Metafer automated capture software and a CCD camera (MetaSystems, Milano, Italy). The frequency of foci per cell were scored in 100 nuclei in at least two independent experiments.

### 2.4. TIF Immunofish Staining

After treatment, cells were seeded on a glass in petri dish and left to grow up the fixation. Slides were treated as described for the immunofluorescence staining, with the same antibodies. After the incubation with the secondary antibody, slides were washes in PBS/Triton X-100 0.05% and then fixed in 4% formaldehyde for 10 min and dehydrated through graded alcohols (70, 80, 100%). Slides and probes (Cy3 linked telomeric peptide nucleic acid (PNA) probe, Panagene, Daejeon, South Korea) were co-denatured at 80 °C for 3 min and hybridized for 2 h at RT in a humidified chamber. After hybridization, slides were washed twice for 15 min in 50% formamide, 10 mM Tris, pH 7.2, and 0.1% BSA followed by three 5-min washes in 0.1 M Tris, pH 7.5, 0.15M NaCl and 0.08% Tween 20. Slides were then dehydrated with an ethanol series and air dried. Finally, slides were counterstained with DAPI (Sigma Aldrich) in Vectashield (Vector Laboratories, Burlingame, CA, USA). Cells, in particular colocalization between γH2AX or 53BP1 foci and telomere, were analyzed using an Axio Imager Z1 microscope (Carl Zeiss, Oberkochen, Germany) equipped with the Metacyte module of the Metafer automated capture software and a CCD camera (MetaSystems, Milano, Italy). The frequency of colocalization dots per cell were scored in 100 nuclei in at least two independent experiments.

### 2.5. Senescence Associated β–Galactosidase Assay

The SA–β-Galactosidase assay was performed using the Senescence β-Galactosidase Staining Kit according to the manufacture’s instruction (Cell Signaling, 9860). The percentage of senescence cells was calculated on about 500 nuclei in at least three independent experiments.

### 2.6. Cell Synchronization Protocol

**Figure 1:**
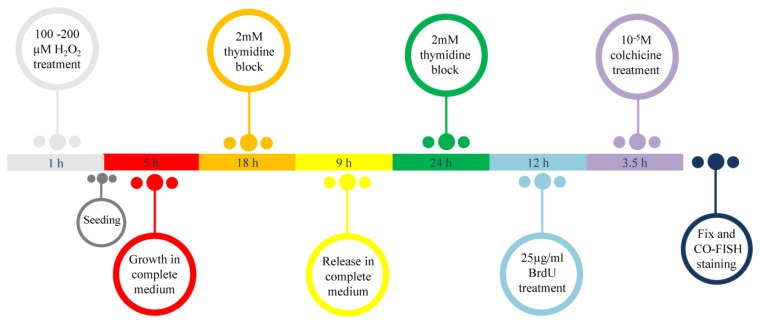
Schematic representation of the cell synchronization protocol and the metaphase preparation timing. After 1 h of treatment with 100 and 200 µM H_2_O_2_, cells were trypsinized and seeded in petri dish. After 5 h, the cells were incubated for 18 h in 2 mM thymidine, released in a complete medium for 9 h and then treated again for 24 h with 2 mM thymidine. Then the cells were treated with 25 µg/mL of BrdU for 12 h and incubated for 3.5 h in 10^−5^ M colchicine. At the end the cells were fixed to obtained metaphases and processed by CO-FISH.

To study telomere replication, MRC-5 cells were synchronized by a double thymidine block. After 1 h treatment with both doses of H_2_O_2_ (100 and 200 µM), cells were trypsinized and seeded in petri dishes at the density of 1 × 10^6^ cells. After 5 h cells were incubated for 18 h in 2 mM thymidine, released in a complete medium for 9 h in S phase after 3 washes with PBS, then treated again for 24 h with 2 mM thymidine and released in S phase after 3 washes in PBS (Figure 1).

### 2.7. Cell Cycle

For cell cycle analysis, after each treatment, 1 × 10^6^ cells were washed twice with PBS and fixed dropwise with ice cold ethanol (70%). DNA staining was performed by rehydrating fixed cells with PBS/0.5% BSA, incubating cells for 30 min at 37 °C in PBS containing 50 µg/mL of propidium iodide (PI) and 150 µg/mL RNase DNase free (type 1-A). Samples were acquired with Cytoflex Flow Cytometer (Beckman Coulter) equipped with a 488 nm laser source. Cell cycle analysis was performed using a CytExpert v2.2 software (Beckman Coulter, Brea, CA, USA). Doublet discrimination was performed by an electronic gate on FL3-Area vs. FL3-Height. Each analysis was performed by acquiring 10,000 events/sample.

### 2.8. BrdU Incorporation

For BrdU incorporation analysis, each sample at the last half hour of time point was pulse 30 min with 10 µM of BrdU and after each time point, cells were fixed and permeabilized with 70% ethanol and the histones were dissociated with 2 M HCl to allow the antibody entry. Acid pH were neutralized by Na_2_B_4_O_7_ 0.1 M pH 8. After extensive washes with PBS/0.5% BSA/0.1% Triton, BrdU positive cells were detected with an anti-BrdU primary antibody diluted 1:100 (DAKO, SA, Glostrup, Denmark) and with an anti-mouse-Alexa 488 conjugated diluted 1:100 (Invitrogen, Life Technologies Corp., Carlsbad, CA, USA). Both antibodies were incubated for 1 h RT in the dark. All samples were counterstained with propidium iodide for DNA content/BrdU biparametric analysis. BrdU percentage was calculated using the CytExpert v2.2 software (Beckman Coulter). Each analysis was performed by acquiring 10,000 events/sample.

### 2.9. Metaphase Preparation and CO-FISH (Chromosome Orientation-FISH) Analysis

After synchronization, cells were released in S phase, washed 3 times with PBS and incubated for 12 h in 5’-bromo-2’-deoxyuridine (BrdU; Sigma Aldrich) at the final concentration of 25 µg/mL. After 3 washes in PBS, cells were incubated for 3.5 h in 10^−5^ M colchicine (Figure 1). Cells were then collected and metaphase spreads were prepared by a standard procedure consisting of treatment with a hypotonic solution (75 mM KCl) for 28 min at 37 °C, followed by fixation in freshly prepared Carnoy solution (3:1 *v/v* methanol/acetic acid). Cells were then dropped onto slides, air dried, and utilized for cytogenetic analysis. CO-FISH, a recombination-based mechanism that analyzed the telomere recombination events between sister chromatids, was performed as previously described by Berardinelli and co-authors [41]. We used first a (TTAGGG)_3_ probe labeled with FITC and then a (CCCTAA)_3_ probe labeled with Cy3 (Panagene, Korea). Images were captured with an Axio Imager Z1 (Carl Zeiss, Germany) equipped with the MSearch module of the Metafer automated capture software and a CCD camera (MetaSystems, Milano, Italy). For each probe unreplicated and replicated telomere was analyzed by counting double and single telomeric signals, respectively. The percentage of either replicated or unreplicated telomeric was calculated on about 3000 chromosomes in three independent experiments.

### 2.10. Data Analysis

We performed the student’s *t*-test for the analysis of the density of telomeric marks in ChIP, for the analysis of foci and TIFs, for the analysis of senescence, and for the analysis of telomeric signals in CO-FISH. Significance was accepted for value *p* < 0.05.

## 3. Results

### 3.1. Oxidative Stress Induces a Reduction in Telomeric Binding Proteins TRF1 and TRF2

**Figure 2:**
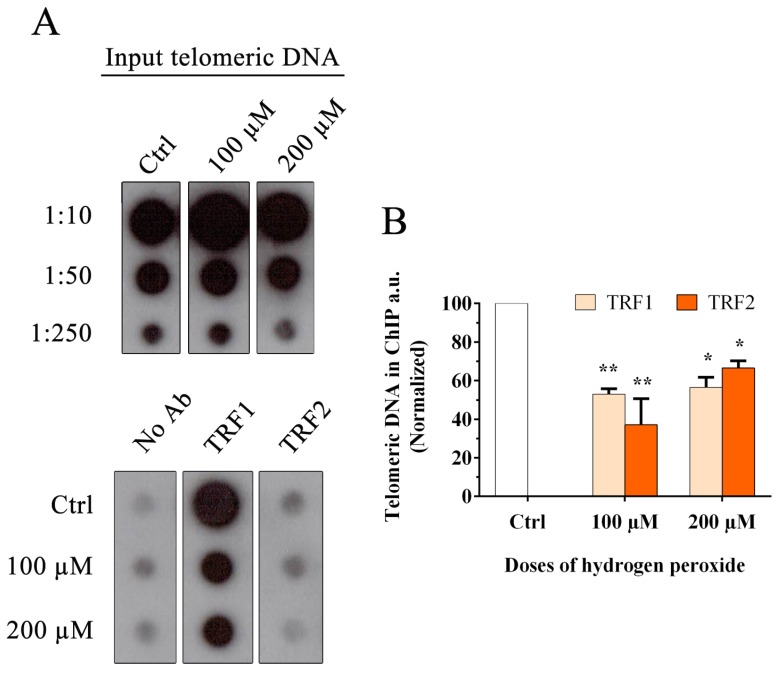
Chromatin immunoprecipitation and telomere dot-blot for TRF1 and TRF2. (**A**) Chromatin immunoprecipitation of MRC-5 cells after H_2_O_2_ treatment (100 µM and 200 µM) with the indicated antibodies (TRF1 and TRF2) and the negative control (No Ab). Telomeric input signals are necessary for the quantification. For this analysis, a 1:10 input was used. (**B**) The histogram represents the data obtained by ChIP analysis at 48 h after treatment with two doses of hydrogen peroxide, 100 and 200 μM. Quantification of the immunoprecipitated telomeric sequences was performed after normalization to telomeric input signals. The data are normalized to the control value and are expressed as a percentage of the total telomeric DNA in arbitrary units (a.u.). The error bars denote the standard errors and were calculated using standard propagation rules. Statistical analysis was performed between treated and control samples. * *p* < 0.05; ** *p* < 0.01 by Student’s *t*-test.

In our previous work we observed that at 24 h after H_2_O_2_ treatment, there was persistent telomeric damage that was not repaired, leading to chromosome instability [21]. Considering previous experiments with modified telomeric oligonucleotides that contain 8-oxoG, in which the authors showed alterations in telomere recognition by TRF1 and TRF2, and regarding the important role of TRF1 and TRF2 in t-loop formation and telomere replication [23,25,26,27], our interest was to understand whether oxidative damage may also influence the binding of telomeric proteins to telomeres in mammalian cells. For this reason, we performed the chromatin immunoprecipitation (ChIP) analysis at 48 h after treatment with two doses of hydrogen peroxide, 100 and 200 µM. Quantification of immunoprecipitated telomeric DNA that binds TRF1 and TRF2 was performed after normalization to the input telomeric signal (Figure 2A). The data shown in Figure 2B indicate a significant reduction at the telomeric level for both TRF1 and TRF2 after hydrogen peroxide treatment. In detail, we observed reductions of 47% and 43.5% for 100 µM and 200 µM H_2_O_2_, respectively, for TRF1 (100 µM, *p* < 0.05; 200 µM, *p* < 0.05), and 63% and 33.5% for 100 µM and 200 µM H_2_O_2_, respectively, for TRF2 (100 µM, *p* < 0.05; 200 µM, *p* < 0.05). These data also indicated a significant reduction of TRF1 and TRF2 at telomeres in vitro.

### 3.2. Oxidative Stress Induces an Increase in the Genomic Damage and an Increase in γH2AX Telomere Dysfunction-Induced Foci (TIFs)

**Figure 3:**
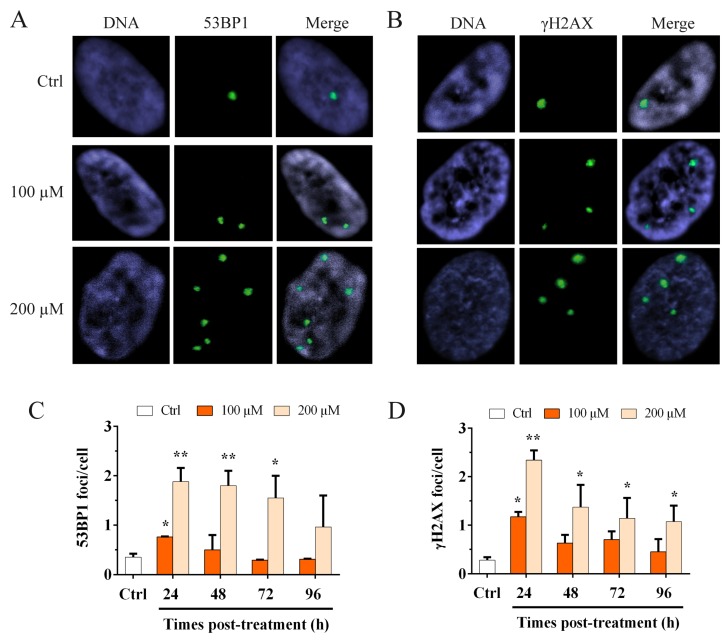
Immunofluorescence staining for 53BP1 and γH2AX foci. Images of MRC-5 cells stained for (**A**) 53BP1 and (**B**) γH2AX. Immunofluorescence staining for 53BP1 and γH2AX was used to detect the activation of the genomic DDR. The columns show the frequency of (**C**) 53BP1 foci per cell and (**D**) γH2AX foci per cell, evaluated after 100 and 200 µM H_2_O_2_ treatment. At 24 h after treatment, we observed a significant increase in the 53BP1 and γH2AX foci for both doses. Conversely, for the higher dose, we showed a significant increase in both 53BP1 and γH2AX at 24 h that significantly persisted for up to 96 h. The bars denote the standard error. Statistical analysis was performed between treated and control samples. * *p* < 0.05; ** *p* < 0.01 by Student’s *t*-test.

The shelterin complex is fundamental for telomere protection and is able to repress the activation of ATM and ATR pathways involved in the DDR response [42]. Considering the data obtained by ChIP for TRF1 and TRF2, we wanted to evaluate the activation of the DDR, not only at the telomere but also at the genomic level, after oxidative stress. To perform this analysis, we performed the immunofluorescence staining for 53BP1 (Figure 3A) and the phosphorylated form (S139) of H2AX (γH2AX) (Figure 3B), and we analyzed the number of foci per cell. The results obtained at different times post H_2_O_2_ treatment are shown in Figure 3C–D. We observed a significant (*p* < 0.05) 2.1-fold increase in the 53BP1 foci number for 100 µM H_2_O_2_ at 24 h after treatment (Figure 3C) that returned to the control value at 96 h after treatment. For the 200 µM H_2_O_2_ treatment we observed a significant (*p* < 0.01) 5.3 and 5.1-fold increase at 24 and 48 h after treatment, respectively, that still persisted significantly (*p* < 0.05) at 72 h after treatment and decrease at 96 h (Figure 3C). For γH2AX foci, we also observed a significant (*p* < 0.05) 4.2-fold increase for 100 µM H_2_O_2_ at 24 h after treatment that deceased in a time-dependent manner up to 96 h. Conversely, for the 200 µM H_2_O_2_ treatment, we observed a significant (*p* < 0.01) 8.3-fold increase in the γH2AX foci at 24 h after treatment, that decreased at 96 h after treatment, although the damage significantly persisted (Figure 3D). These results showed that, for the higher dose of hydrogen peroxide, the genomic DNA damage persisted for up to 96 h after treatment.

**Figure 4:**
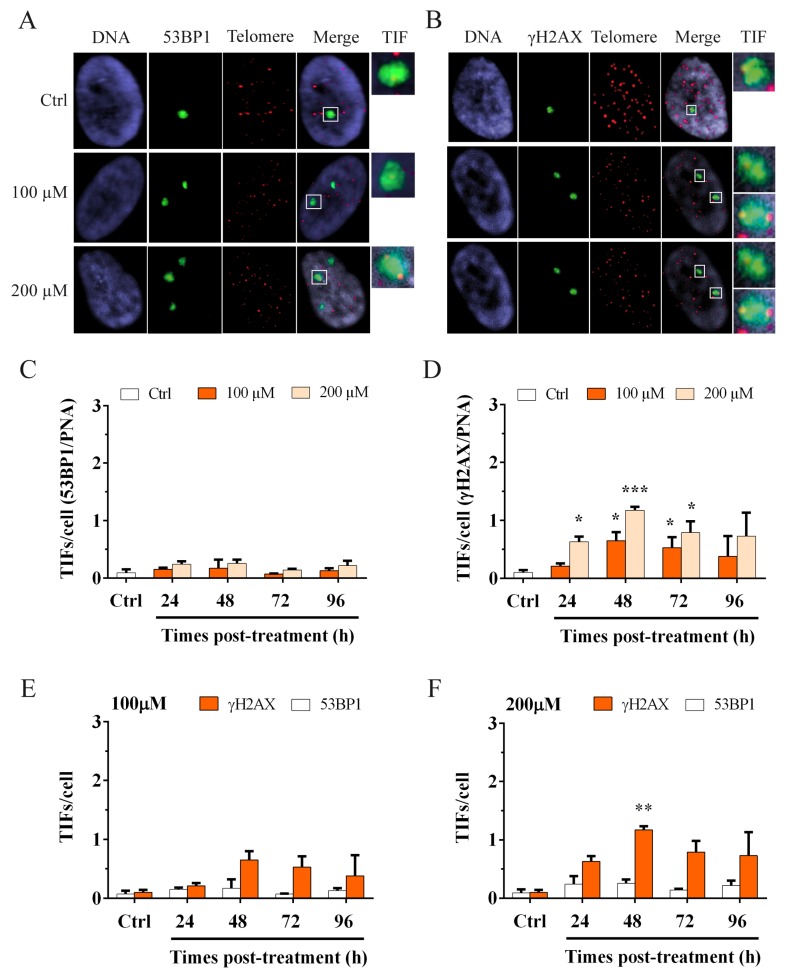
ImmunoFISH staining to detect telomere dysfunction-induced 53BP1 and γH2AX foci (TIFs). Images of MRC-5 cells stained for (**A**) 53BP1 foci and the telomeric PNA probe and (**B**) γH2AX foci and the telomeric PNA probe. The colocalization of both signals (green and red spots) indicates the presence of a telomere dysfunction-induced focus (TIF). More than a one telomeric signal can colocalize with 53BP1 or γH2AX foci (as shown in Figure 4A and Figure 4B-TIF). TIF (last boxes) are enlarged to show the colocalization between 53BP1 and telomeres (**A**) and between γH2AX and telomeres (**B**). ImmunoFISH staining was used to detect the activation of the DDR at telomeres. The graphs represent the results of telomeric damage after oxidative stress at different times post-treatment. Two markers of DNA damage were used in the analysis. (**C**) 53BP1 TIFs. The columns show data obtained by colocalization between 53BP1 foci and telomeres per cell. We observed no differences in treated and untreated samples for both doses of hydrogen peroxide at different times post-treatment. (**D**) γH2AX TIFs. The columns show data obtained by colocalization between γH2AX foci and the telomeres per cell. We observed a significant increase (*p* < 0.05) of foci at 48 h after the 100 µM treatment that persisted at 72 h. For 200 µM H_2_O_2_, we observed a significant increase at 24 h after treatment, which significantly increased (*p* < 0.001) at 48 h and still significantly persisted (*p* < 0.05) at 72 h after treatment. Statistical analysis was performed between treated and control samples. The bars denote the standard error. Statistical analysis was performed between treated and control samples for both 53BP1 and γH2AX TIFs/cell. * *p* < 0.05; *** *p* < 0.001 by Student’s *t*-test. Comparison between 53BP1 and γH2AX TIFs at (**E**) 100 µM H_2_O_2_ and (**F**) 200 µM H_2_O_2_. The graphs show the higher increase of γH2AX TIFs with respect to 53BP1 TIFs, especially at 48 h after 200 µM H_2_O_2_ treatment (**F**), in which this difference was statistically significant (*p* < 0.01). The bars denote the standard error. Statistical analysis was performed between 53BP1 and γH2AX TIFs/cell. ** *p* < 0.01 by Student’s *t*-test.

To focus attention on telomere dysfunction, however, it is important to evaluate the activation of the DDR specifically at the telomeric level. With this aim, we performed immunoFISH staining to detect telomere dysfunction-induced foci (TIFs) and the colocalizations of the DNA damage markers 53BP1 (Figure 4A) or γH2AX (Figure 4B), with telomeres. The results obtained are shown in Figure 4C,D. We observed no change in the frequency of 53BP1 TIFs at different times after treatment for both doses of hydrogen peroxide (Figure 4C). Conversely, we observed a significant increase (*p* < 0.05) in γH2AX TIFs at 48 h after treatment with 100 µM H_2_O_2_ that significantly persisted (*p* < 0.05) at 72 h after treatment and then decreased at 96 h (Figure 4D). For 200 µM H_2_O_2_, we observed a significant increase (*p* < 0.05) in γH2AX TIFs at 24 h after treatment that further increased significantly after 48 h (*p* < 0.01). In this case, the frequency ranged from 0.65 for 100 µM to 1.17 for 200 µM. At 72 h after treatment, we observed a statistically significant (*p* < 0.05) persistence of γH2AX TIFs that decreased at 96 h (Figure 4D). Considering the divergent results obtained by the analysis of the two DNA damage markers at telomeres and taking into account their different roles in the DDR, we performed another analysis to understand what type of damage occurs at telomeres after oxidative stress.

### 3.3. Hydrogen Peroxide Treatment Induces a Telomeric Replication Fork Block Rather Than a DSB

Oxidative damage can induce replication fork arrest and γH2AX is activated immediately thereafter [9,43]. The evaluation of telomere dysfunction-induced foci (TIFs) was performed to understand the type of damage that occurs at telomeres after oxidative stress. Using the data shown in Figure 4C,D, we split the results for the two doses, 100 and 200 µM (Figure 4E,F, respectively). The results indicate a higher number of γH2AX TIFs (orange columns) with respect to 53BP1 TIFs (white columns) for all times and at both doses of hydrogen peroxide. A statistically significant difference (*p* < 0.01) was observed at 48 h after 200 µM H_2_O_2_ treatment (Figure 4F), where the induction of telomere foci ranging from 0.26 for 53BP1 to 1.17 for γH2AX (Figure 4F). These results suggest that oxidative stress-inducing γH2AX causes a stall of the replication fork rather than a DSB at telomeres.

### 3.4. Oxidative Stress Induces Premature Senescence

**Figure 5:**
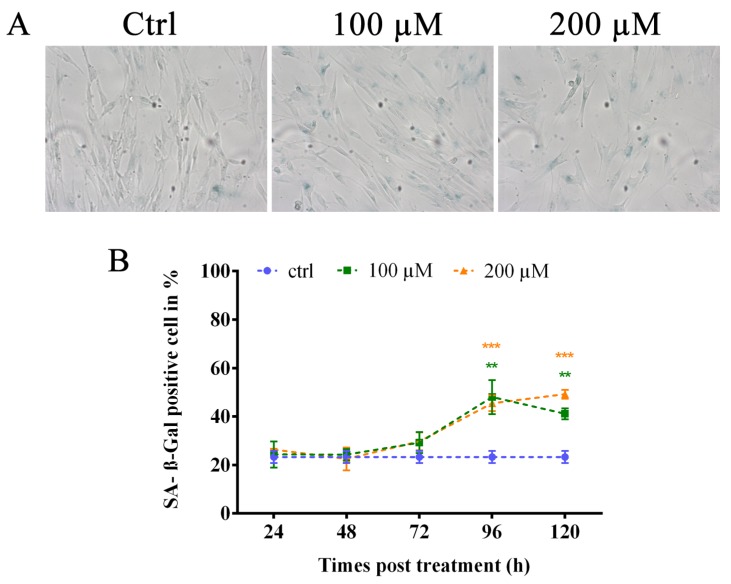
Analysis of SA-β-galactosidase-positive cells. (**A**) Representative images of positive SA-β-Gal-positive cells. (**B**) The graph represents data obtained from the analysis of the percentage of SA-β-galactosidase-positive cells. Control values are shown as the mean of the controls analyzed at different times. We observed a significant increase in the percentage of SA-β-Gal-positive cells at 96 h after treatment at both doses of hydrogen peroxide that persisted at 120 h after treatment. The error bars denote the standard error. ** *p* < 0.01; *** *p* < 0.001 by Student’s *t*-test.

Due to the persistence of TIFs under our experimental condition and considering that different authors state that sublethal hydrogen peroxide treatment could induce a senescence-like growth arrest, we studied the induction of premature senescence by oxidative stress [16,18,44,45]. We performed an analysis of senescence by evaluating the activity of senescence-associated β-galactosidase (Figure 5A) at different times post-treatment, starting from 24 to 120 h. The results showed a significant increase (*p* < 0.01 for 100 µM and *p* < 0.001 for 200 µM) of SA-β-Gal-positive cells at 96 h after treatment with both doses of hydrogen peroxide (48% and 45.45% for 100 and 200 µM, respectively) that significantly persisted (*p* < 0.01 for 100 µM and *p* < 0.001 for 200 µM) at 120 h after treatment (Figure 5B). These data confirm the induction of stress-induced premature senescence in human primary fibroblasts after hydrogen peroxide treatment.

### 3.5. Oxidative Stress Induces a Genomic Reduction of Replication Rate

**Figure 6:**
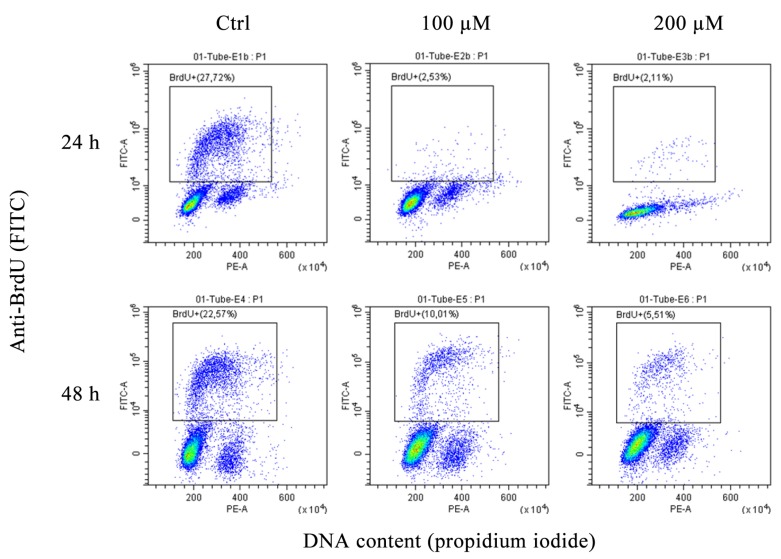
Cytometric analysis of BrdU incorporation. The incorporation of BrdU was used to detect the cell proliferation status by flow cytometry. In this figure, biparametric dot plots of control or H_2_O_2_-treated MRC-5 cells show that this treatment, at 24 h (**A**), completely arrested cell proliferation. Which was slightly recovered at 48 h (**B**). The magnitude of this cell cycle arrest effect is dose related. The box gate on dot plots represents BrdU-positive cells.

Based on the above results, to investigate the hypothesis that replication fork arrest at telomeres is induced by oxidative stress, we performed different analyses to identify a decreased rate of DNA replication at the ends of chromosomes. First, we performed an analysis of the genomic replication rate by adding 5’-bromo-2’-deoxyuridine (BrdU), a thymidine analog that is casually incorporated into the newly synthesized DNA strand during replication, to the culture medium at a half an hour before the fixing time. Using an antibody against BrdU, we detected the number of cells in S-phase of the cell cycle. Figure 6 represents a biparametric dot plot cytometric analysis that measures the percentage of BrdU-positive cells and their DNA content by propidium iodide staining. BrdU-positive cells are enclosed on a box gate, and their percentages were calculated. This experiment clearly shows that at 24 h after treatment with 100 and 200 µM H_2_O_2_, S-phase completely disappeared, considering that 27.72% ± 2.5% (mean ±SEM) BrdU-positive cells in the control sample, decreased to 2.53% ± 0.8% and 2.11% ± 1.2% in response to 100 and 200 µM, respectively (Figure 6A); the residual cells were almost all blocked at the G1 phase. At 48 h, S-phase cells ranged from 22.57% ± 1.7% in the control samples to 10.01% ± 2.2% and 5.51% ± 1.1% in the 100 and 200 µM-treated samples, respectively (Figure 6B), indicating a global growth delay that is probably due to the treatments.

### 3.6. Oxidative Stress Induces Replication Fork Arrest at Telomerse

**Figure 7:**
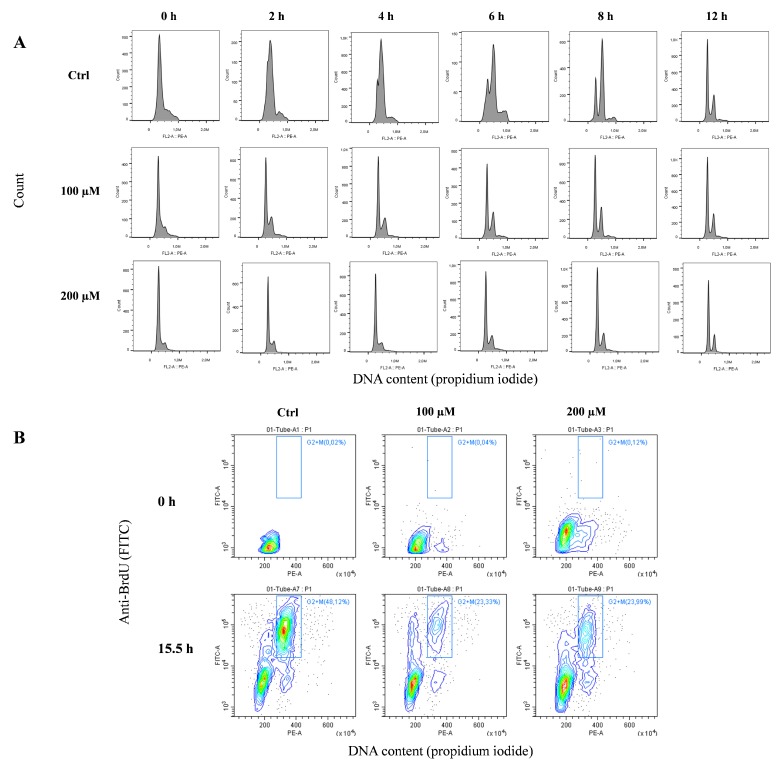
Analysis of the cell cycle after thymidine release and BrdU incorporation. (**A**) Monoparametric analysis of the DNA content was used to identify the timing of the S-phase after thymidine cell cycle synchronization. The histograms show that the S wave of the synchronization progresses towards G2 in untreated cells. In treated cells the slow progress of the S-phase makes synchronization less noticeable, but G2 is more evident at 12 h after thymidine release. (**B**) The incorporation of BrdU was used to detect G2/M phase accumulation and the relative metaphase block, with a treatment schedule of 12 h of thymidine followed by 3.5 h of colchicine (15.5 h).

Before analyzing the replication fork arrest at telomerse through CO-FISH (chromosome orientation fluorescence in situ hybridization) (see below), it was important to understand the timing of the S-phase of the cell cycle. To better demonstrate the replication block, we synchronized the cells with a double treatment of thymidine (see the Materials and Methods section). This pyrimidine deoxynucleoside synchronizes cells at the G1/S boundary. After double treatment with thymidine, we performed a cell cycle analysis to evaluate the synchronization rate of cells and the timing of the S-phase. Figure 7A shows the monoparametric distribution of the DNA content after thymidine release in complete medium following thymidine block for different hours (from 0 to 12 h). In untreated cells, the progression of the S-phase wave starts at 2 h, and at 12 h, the normal cell cycle is restored. In contrast, in peroxide-treated cells, the exit from G1 is very slow, as demonstrated by the S-phase wave being very low at all times points.

Considering these results, to better understand the progression of the S-phase and its G2/M accumulation, we treated the cells with BrdU for 12 h after synchronization, followed by 3.5 h of colchicine (15.5 h). Figure 7B shows the analysis of the cell cycle and BrdU incorporation. This biparametric plot shows that in untreated cells, colchicine induces an evident accumulation of cells in the G2/M phase (approximately 50%). In contrast, peroxide-treated cells are blocked at the G1/S phase and only 23–24% are able to reach the G2/M phase.

**Figure 8:**
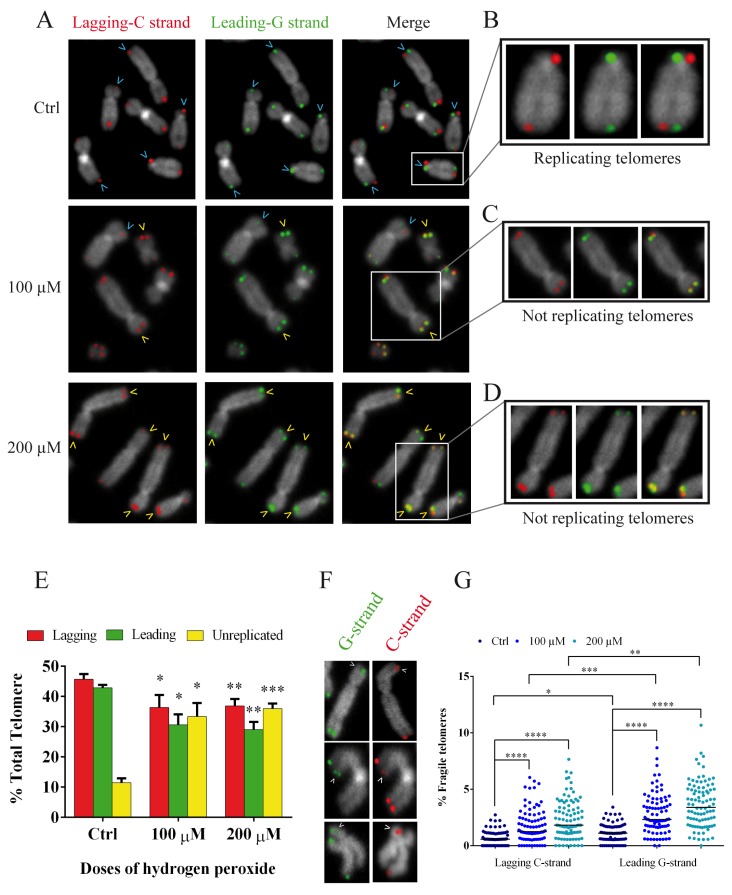
Chromosome Orientation-FISH to study replication at telomere. (**A**) Representative image of control and treated MRC-5 chromosomes, labeled by telomere-specific C- and G-rich probes. In detail red spots indicate the lagging C-strand, and green spots indicate the leading G-strand. The presence of both signals indicates unreplicated telomeres highlighted with yellow arrows, while blue arrows indicate replicated telomeres that displayed only one signal on each chromatid. Three enlarged chromosomes with replicated (**B**) and unreplicated telomere are shown to the right of the picture (**C**,**D**). The quantification of the % of replicated (both leading and lagging) and unreplicated telomeres is shown in the graph (**E**), demonstrating a significant decrease in replicated telomeres and an increase of unreplicated telomeres in 100 and 200 µM H_2_O_2_-treated samples with respect to the controls. The error bars denote the standard error. (**F**) Representative images of chromosomes with fragile sites for both the leading and lagging strands. The quantification of fragile telomeres is shown in (**G**), showing an increase in the percentage of fragile telomeres for the leading and lagging strands after treatment with both doses of hydrogen peroxide. The lines represent the median. Statistical analysis was performed between treated and control samples. * *p* < 0.05; ** *p* < 0.01; *** *p* < 0.001 by Student’s *t*-test.

The above results on synchronized fibroblasts allowed us to choose the best time for the analysis of replication fork arrest specifically at telomeres because we had a high number of mitoses and the guarantee of analyzing cells that had passed the S-phase. In fact, due to the results mentioned above, we performed a CO-FISH analysis following the same treatment with BrdU and colchicine (12 + 3.5) after synchronization with thymidine (Figure 7B). CO-FISH technique, using two different probes labeled for G- and C-rich telomeric sequences, is able to discriminate the telomeric leading and lagging strands (Figure 8A) [46,47,48]. When BrdU is introduced during the S-phase, it is incorporated into the daughter strand; treatment with UV and subsequent digestion with exonuclease III disrupts the new BrdU-incorporated strand, yielding a single-stranded chromatid that is available for hybridization with complementary probes labeled with different fluorochromes [46,47,49]. In contrast, unreplicated telomeres do not incorporate BrdU. Instead, they maintain both strands and allow the hybridization of leading and lagging strands, showing a double signal in each chromatid. In this way we can discriminate, in the same metaphase, the replicated (one signal for each chromatid) and unreplicated (both signals for each chromatid telomere) telomeres (Figure 8A–D). The results obtained by CO-FISH analysis are shown in Figure 8E. We observed a significant reduction of replicated telomeres in the treated samples compared to the control samples for both lagging and leading strands and for both doses of hydrogen peroxide; on the other hand, we observed a significant increase of unreplicated telomeres in treated samples starting from control values of 11.5% up to 33.4 and 36% for 100 and 200 µM, respectively (Figure 8E).

In addition, the deletion of TRF1 induces the formation of multitelomeric signals, also known as fragile telomeres, which are markers of hindered replication machinery. These aberrant telomeric structures are often referred to as telomere doublets (Figure 8F). Considering our results demonstrating a decrease in the amount of TRF1 in the hydrogen-peroxide treated samples, we investigated the presence of telomere doublets at 48 h after both 100 and 200 µM H_2_O_2_ treatments (Figure 8G). Using the CO-FISH technique as previously described, we were able to discriminate telomere doublets for both the leading and lagging strands (Figure 8F). Our results showed a significant increase (*p* < 0.001) in the percentage of fragile telomeres, for both the leading and lagging strands. In detail, the lagging C strand increased from 0.7% in the control sample to 1.77% for 100 µM and 2.36% for 200 µM, and the leading G strand increased from 1.12% in the control sample to 2.82 for 100 µM and 3.36 for 200 µM (Figure 8G). It is evident that this increase is significantly higher for the leading G strand with respect to the same sample of the lagging C strand.

These results support our hypothesis that replication fork arrest is induced by oxidative stress.

### 3.7. Oxidative Stress Induces an Increase in the Heterochromatin Mark H3K9me3

**Figure 9:**
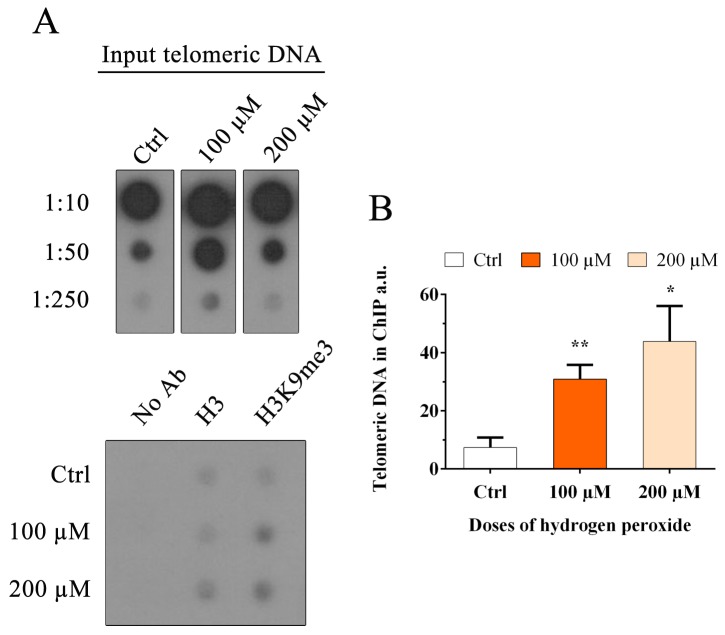
Chromatin immunoprecipitation and telomere dot-blot for H3K9me3. (**A**) Chromatin immunoprecipitation of MRC-5 cells after H_2_O_2_ treatment (100 µM and 200 µM) with the indicated antibody (H3K9me3) and negative control (No Ab). Telomeric H3 signals are necessary for quantification. No alterations in the amount of H3 were observed after treatment. For this analysis, a 1:250 input ratio was used. (**B**) The histogram represents data obtained by ChIP analysis at 48 h after treatment for both doses of hydrogen peroxide, 100 and 200 μM. The quantification of immunoprecipitated telomeric sequences was performed after normalization to the telomeric H3 signal. The data are expressed as a percentage of the total telomeric DNA in arbitrary units (a.u). The results showed a significant increase in H3K9me3 (100 μM, *p* < 0.01; 200 μM, *p* < 0.05) at both doses. The error bars denote the standard errors. Statistical analysis was performed between treated and control samples. * *p* < 0.05; ** *p* < 0.01 by Student’s *t*-test.

Recent studies have demonstrated that genomic epigenetic changes could be associated with replication fork arrest; furthermore, oxidative stress induced by H_2_O_2_ has been associated with (i) the recruitment of DNA methyltransferase 1 to damaged chromatin, (ii) the increase in global histone methylation and (iii) the modification of active chromatin to a repressive form [36,37,38,39,50]. No evidence is present in the literature on specific telomeric histone methylation. In this study, we evaluated the amount of H3K9me3, a marker for heterochromatin, at telomeres. ChIP analysis was performed as the best method used to detect the modification status of histones associated with a specific region. The quantification of immunoprecipitated telomeric DNA with H3K9me3 modification was performed after normalization to the telomeric H3 signal (Figure 9A). The results obtained at 48 h after treatment with hydrogen peroxide are shown in Figure 9B. We observed a significant increase in H3K9me3 after hydrogen peroxide treatment from 0.07 in the control sample to 0.3 and 0.44 in 100 and 200 µM H_2_O_2,_ respectively (100 µM, *p* < 0.01; 200 µM, *p* < 0.05), indicating telomeric heterochromatinization after treatment.

## 4. Discussion

We are subjected daily to endogenous or exogenous sources of ROS that can accumulate in cellular compounds and alter cellular functions. Additionally, in many clinical disorders (e.g., diabetes and neurodegenerative diseases) or under normal conditions such as aging, ROS are important for or are produced during pathogenesis (reviewed in reference [51]). Thus, there is great interest in studying the damage induced by oxidative stress and understanding the mechanisms that lead to oxidative damage and, in some cases, to pathologies.

DNA represents one of the most vulnerable biological molecules to oxidative damage; in particular telomeres represent the preferential target of oxidative stress due to their high content in guanine residues and their lower efficiency in DNA damage repair [21,52].

In our previous study we demonstrated persistent telomeric oxidative damage (8-oxoG) after acute treatment with hydrogen peroxide that gives rise to chromosome instability related to telomere shortening/dysfunction. In particular, we observed micronuclei (MN), nucleoplasmic bridges (NPBs) and nuclear buds (NBUDs), together called abnormal nuclear morphologies (ANMs), which increased at 48 h after treatment [21]. Several authors have studied the effects of oxidative stress on telomeres but in this study, we focused on obtaining a deeper understanding of the molecular mechanisms that lead to telomere shortening/dysfunction due to the presence of oxidative stress damage.

The telomere is a particular specific chromosome region with a peculiar organization and, consequently, a proper DNA damage response. Understanding telomeric oxidative damage and how telomeres react to this lesion could provide us with a wide view of the mechanisms and consequences of telomere oxidative damage. First, we focused on the analysis of telomere structure and shelterin proteins TRF1 and TRF2 because of their predominant role in telomere stability. Previous data obtained on oligonucleotides that contain telomeric sequences modified with 8-oxoG have reported that this lesion might interfere with telomere function by inhibiting the association of TRF1 and TRF2 proteins with telomeric sequences [23]. Because few data are available on oligonucleotides and no analysis was performed in vitro or in vivo, we investigated the possibility of the involvement of these telomeric proteins in oxidative stress-induced telomeric damage in human cells. Using chromatin immunoprecipitation (ChIP), we analyzed the amount of TRF1 and TRF2 that binds specifically to the telomeric region after hydrogen peroxide treatment. Our results indicated a significant reduction in TRF1 and TRF2 at telomeres at 48 h after treatment. This reduction, confirming data obtained by Opresko and coauthors on oligonucleotides, suggests that the persistent telomeric 8-oxoG previously reported could also inhibit TRF1 and TRF2 telomere binding in the cellular system [21,23].

In addition, other studies have demonstrated that TRF1 and TRF2 are fundamental for telomere protection because the proteins are involved in proper telomere replication and T-loop formation, respectively [25,26,27]. The presence of these proteins inhibits the activation of DNA damage signaling at telomeres [53]. In fact, previous studies, performed with TRF1 knockout mouse embryonic fibroblasts (MEFs) have demonstrated the activation of DDR and the appearance of 53BP1 and γH2AX foci at telomeres (TIFs) [26]. It was also hypothesized that TRF1, which is important for telomere replication, was responsible for the replication stress that activated the DDR [27]. Other studies have demonstrated that the deletion of TRF2 from MEFs made telomeres dysfunctional, promoting the activation of the DDR and inducing end-to-end fusion [54,55,56]. These results correlate very well with our data showing that TRF2 reduction could be related to the increase in NPBs as previously reported [21].

To study the consequences of the observed TRF1 and TRF2 reduction on telomeres, we compared genomic and telomeric damage by immunostaining for two markers widely used in the detection of DNA damage, 53BP1 and γH2AX. 53BP1 is activated in DSB repair, while H2AX is phosphorylated not only when DSBs occur but also when the replication fork arrests [9]. The analysis of 53BP1 and γH2AX foci in the nucleus showed a significant increase in DNA damage at 24 h after treatment with both doses of hydrogen peroxide, indicating the presence of DSBs in the genome that are resolved at subsequent times, except for at the higher dose, in which damage persisted for up to 96 h. In contrast, immunoFISH performed specifically on telomeres showed very interesting results. In fact, data obtained by analyzing the colocalization of the DNA damage markers 53BP1 and γH2AX with telomeres showed differences between the two: telomeric 53BP1 foci did not change from untreated to treated samples at any time post-treatment. Conversely, for telomere/γH2AX, there was, for the lower dose, a significant increase at 48 h after treatment that persisted for up to 72 h; for the higher dose, the significant increase at 24 h further increased at 48 h after treatment and persisted for up to 72 h. This persistent telomeric damage that causes a continuous DDR could induce cellular senescence. Considering our results on the presence of TIFs at longer times post-treatment, we performed an analysis of senescence. We observed a significant increase in senescence-positive cells from 96 h after hydrogen peroxide treatment that persisted for 120 h after oxidative stress induction. These data confirm the data from previous studies showing that oxidative stress-induced premature senescence is most likely induced by telomere dysfunction. Therefore, the telomere restoration observed from 72 h could be due to a selective process in favor of cells with longer, functional telomeres that escape senescence. Then, taking into account the role of γH2AX in replication stress and considering the TRF1 reduction certainly involved in telomere replication [26,27], we suppose that the 8-oxoG induced by acute oxidative stress leads to a block of the replication fork rather than a DSB at telomeres. The idea that oxidative stress interferes with the replication fork at telomeres had already been hypothesized by von Zgliniki and coauthors [57], but nobody has ever evaluated the stall of the replication fork at telomeres, which in our opinion would be the definitive demonstration to explain the telomere shortening induced by oxidative stress. To this end, we first tested the replication rate of MRC-5 cells treated with hydrogen peroxide. Using a cytofluorimetric assay and taking advantage of BrdU incorporation during the S-phase of the cell cycle, we observed a significant reduction in the S-phase at 24 h after treatment that partially recovered at 48 h after 100 and 200 µM H_2_O_2_ treatment, indicating a general delay in the S-phase most likely due to treatment.

Because our goal was to specifically test the replication of telomeres after acute oxidative stress, assuming that human telomeres replicated throughout the S-phase of the cell cycle and to analyze a larger number of replicating cells that exceeded the S-phase, we synchronized human primary fibroblasts [58,59]. For different purposes, many authors have synchronized immortalized cells or tumor cells but only a few studies are available on normal fibroblasts, probably because of the difficulties in performing the synchronization and S-phase labeling in these cells [60,61,62,63]. Our aim was to verify the hypothesis of telomeric replication stress in oxidative stress-treated telomerase negative human primary fibroblasts, with functional checkpoints as one of the main goals of this study. After several attempts, we managed synchronization at the G1/S boundary, and to evaluate the timing of the S-phase, we fixed the cells every two hours until 12 h after the thymidine release. Considering the time of the S-phase and to analyze the telomeres replication during the 12 h, we treated the cells with BrdU during the entire S-phase (12 h) and added colchicine for another 3.5 h, accumulating the highest number of mitoses to study the telomeric replication fork by CO-FISH. In fact, the CO-FISH technique allowed us to discriminate replicated telomeres that showed only one telomeric signal for each chromatid, from unreplicated telomeres that showed both telomeric signals for each chromatid (see results, Section 3.6 on oxidative stress and replication fork arrest). Our results showed a significant reduction of leading and lagging replicated telomeres, probably due not only to the presence of 8-oxoG in the parental G-strand but also to the incorporation of oxidized nucleotides from pools of dNTP precursors in the newly synthetized leading strand [64]. Additionally, a significant increase in unreplicated telomeres was observed in H_2_O_2_-treated samples compared to the control; all these results highlighted the replication block at telomeres due to oxidative stress.

To further support this conclusion, since the TRF1 deletion is also responsible for fragile telomeres, another marker of replication machinery block, we analyzed multitelomeric signals [26,27]. The data obtained from the analysis of chromosome ends showed a significant increase in fragile telomeres after H_2_O_2_ treatment; even when the increase in fragile telomeres was higher in the G strand, fragile telomeres were significantly increased in both strands. This result supports the idea that G-oxidation occurs in the pool of nucleotides and provides further evidence that a block of the replication fork at telomeres occurs. In our opinion, it is specifically the 8-oxoG modification that is responsible for the observed replication block.

**Figure 10:**
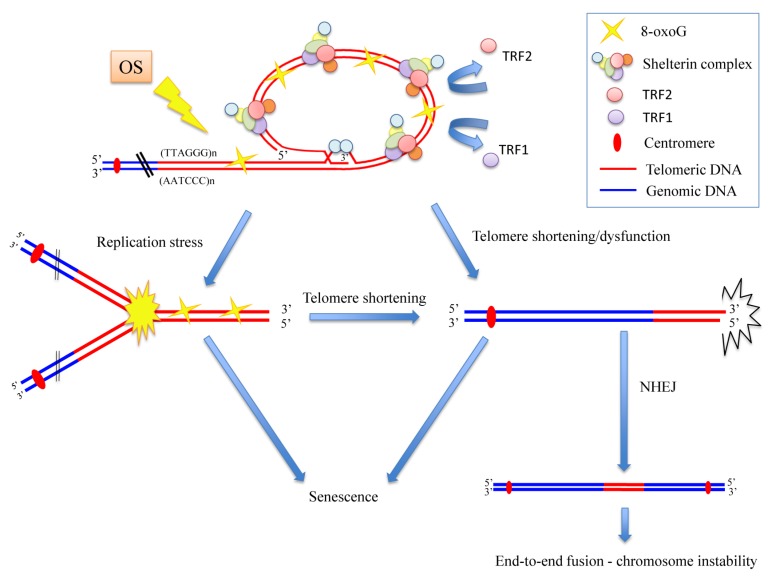
Model of how oxidative stress (OS) induces chromosome instability mainly by telomere damage. We suppose that 8-oxoG, induced by oxidative stress, is responsible of TRF1 and TRF2 reduction. The decreased binding of these telomeric proteins is responsible of replication fork arrest and telomere shortening/dysfunction that in turn cause senescence and chromosome instability, especially end-to-end fusions. NHEJ (non-homologous end joining).

Our idea was that oxidative stress induces 8-oxoG, which is the principal and less repaired lesion at the telomere. This base modification is responsible for the TRF1 and TRF2 reduction. The decreased binding of these telomeric proteins, usually involved in end-protection and efficient replication at telomeres, is responsible for replication fork arrest, leading to telomere shortening/dysfunction, which in turn results in chromosome instability, especially end-to-end fusions, growth delay and senescence (Figure 10) (see also our previous results on Coluzzi et al., [21]).

In conclusion, the presence of stalled replication forks is the missing step between oxidative damage induction and SSB accumulation at telomeres that, in the end, will lead to telomere shortening.

To obtain a general understanding of the modifications induced by oxidative stress at telomeres, our last aim of this study was to examine the epigenetic status of telomeres after oxidative stress. Telomeres have a characteristic heterochromatin structure defined by the shelterin complex and histone modification marks that contribute to proper telomere functioning [13,14]. Few studies have been carried out on the relationship between telomeric heterochromatin structure and oxidative stress. Recently, different authors reported at the genomic level an increase in DNA methylation and global histone methylation marks after oxidative stress, in particular repressive histone marks (H3K4me3 and H3K9me3) [35,36,37,65]. No data are available non histone modifications on chromatin conformation changes, specifically at telomeres after oxidative stress. Thus, we wanted to understand whether epigenetic telomeric changes occur after H_2_O_2_ treatment. To this end, the amount of telomeric H3K9me3 was analyzed by ChIP. Our results showed a significant increase in this marker at telomeres at 48 h after H_2_O_2_ treatment. Because of the involvement of this histone modification in chromatin heterochromatinization [33], these data could be interpreted as a more condensed status of telomeric chromatin after acute oxidative stress. Assuming that a situation similar to that observed at the genomic level occurs at telomeres and considering the previous studies at the genomic level in which authors demonstrated a role for heterochromatinization induced by oxidative stress in gene silencing, we could hypothesize a similar effect on the telomere region [35,36]. In fact, a mechanism called the Telomere Position Effect (TPE) represents the ability of telomeres (heterochromatin environment) to silence nearby genes, for example in the subtelomeric region [66,67]. The mechanism that regulates this process is poorly understood in humans; controversial evidence has been reported in the literature. TPE could be related to telomere lengthening, but recent studies have indicated that the silencing of genes near the telomeric region is strictly associated with short telomeres or the total absence of telomere repeats in two different cases of patients affected the Ring17 syndrome [68,69,70]. We speculate that oxidative stress, through higher chromatin condensation, could influence the expression of subtelomeric genes, but this phenomenon should be further investigated.

Another hypothesis on the effects of telomeric chromatin epigenetic modification could be that if on the one hand, oxidative stress damage induces telomere shortening, then, on the other hand, the more condensed status of telomeric chromatin could be due to a link between replication stress and heterochromatin formation, as some authors have recently reported [71,72,73]. Generally, in response to DNA damage, chromatin undergoes global decondensation. Paradoxically, some studies have demonstrated that heterochromatin factors, which are able to induce the trimethylation of H3K9, could accumulate at damaged sites and eventually at sites of replication stress [74,75]. Although further studies are necessary to better understand the role of this crosstalk, this phenomenon could explain our results showing that the stalling of the replication fork could be related to the heterochromatinization of telomeric chromatin we observed after telomeric oxidative damage. Furthermore, because previous studies have demonstrated that the heterochromatin at dysfunctional telomeres is permissive for non-homologous end joining (NHEJ) [76], we hypothesize that the increase of NPBs observed after oxidative stress in our previous study could be due to the increased heterochromatinization responsible for NHEJ activation, which is specifically correlated with telomere fusion (Figure 10) [21,76].
